# Identification and Characterization of a Novel Salt-Tolerant Esterase from the Deep-Sea Sediment of the South China Sea

**DOI:** 10.3389/fmicb.2017.00441

**Published:** 2017-03-23

**Authors:** Yi Zhang, Jie Hao, Yan-Qi Zhang, Xiu-Lan Chen, Bin-Bin Xie, Mei Shi, Bai-Cheng Zhou, Yu-Zhong Zhang, Ping-Yi Li

**Affiliations:** ^1^State Key Laboratory of Microbial Technology, Marine Biotechnology Research Center, Institute of Marine Science and Technology, Shandong UniversityJinan, China; ^2^Laboratory for Marine Biology and Biotechnology, Qingdao National Laboratory for Marine Science and TechnologyQingdao, China

**Keywords:** esterase, salt-tolerance, deep-sea sediment, metagenomics, basic residues

## Abstract

Marine esterases play an important role in marine organic carbon degradation and cycling. Halotolerant esterases from the sea may have good potentials in industrial processes requiring high salts. Although a large number of marine esterases have been characterized, reports on halotolerant esterases are only a few. Here, a fosmid library containing 7,200 clones was constructed from a deep-sea sediment sample from the South China Sea. A gene *H8* encoding an esterase was identified from this library by functional screening and expressed in *Escherichia coli*. Phylogenetic analysis showed that H8 is a new member of family V of bacterial lipolytic enzymes. H8 could effectively hydrolyze short-chain monoesters (C4–C10), with the highest activity toward *p*-nitrophenyl hexanoate. The optimal temperature and pH for H8 activity were 35°C and pH 10.0, respectively. H8 had high salt tolerance, remaining stable in 4.5 M NaCl, which suggests that H8 is well adapted to the marine saline environment and that H8 may have industrial potentials. Unlike reported halophilic/halotolerant enzymes with high acidic/basic residue ratios and low pI values, H8 contains a large number of basic residues, leading to its high basic/acidic residue ratio and high predicted pI (9.09). Moreover, more than 10 homologous sequences with similar basic/acidic residue ratios and predicted pI values were found in database, suggesting that H8 and its homologs represent a new group of halotolerant esterases. We also investigated the role of basic residues in H8 halotolerance by site-directed mutation. Mutation of Arg195, Arg203 or Arg236 to acidic Glu significantly decreased the activity and/or stability of H8 under high salts, suggesting that these basic residues play a role in the salt tolerance of H8. These results shed light on marine bacterial esterases and halotolerant enzymes.

## Introduction

Lipolytic enzymes, including esterases and lipases, are involved in catalyzing the hydrolysis and synthesis of esters. Esterases usually hydrolyze water-soluble short-chain monoesters, while lipases prefer water-insoluble long-chain triglycerides (Jaeger et al., 1999). Marine lipolytic enzymes play an important role in marine organic carbon degradation and cycling. A large number of active microbial lipolytic enzymes have been discovered from surface and deep-sea seawater (Chu et al., 2008; Fang et al., 2014), hydrothermal vents (Placido et al., 2015), and marine sediments (Li et al., 2014), suggesting their potential roles in marine ecosystems.

Marine environments usually contain ∼3.5% (w/v) NaCl, and in some salterns, the salinity can even reach as high as 37% (w/v) (Ventosa et al., 2014). Many microbial enzymes of marine origin have evolved to be halotolerant or halophilic. Several halotolerant or halophilic lipolytic enzymes have been discovered from marine environments, including a halotolerant esterase (Est10) from *Psychrobacter pacificensis* (Wu et al., 2013), a halophilic esterase (LipC) from *Haloarcula marismortui* (Rao et al., 2009), a halotolerant esterase (ThaEst2349) from *Thalassospira* sp. (De Santi et al., 2016), and a halophilic lipase (LipBL) from *Marinobacter lipolyticus* (Perez et al., 2011). Studies on halotolerant/halophilic lipolytic enzymes and other halophilic enzymes show that these proteins have a significant increase in negatively charged acidic amino acid residues over their surfaces, which may form protective hydrated ion network and promote the adaption of the protein to salinity (Oren et al., 2005). The increase in acidity over the surface also prevents the aggregation of proteins (Elcock and Mccammon, 1998). However, there is also a report showing that an increase in positively charged basic residues on the enzyme surface may contribute to the adaption of an endonuclease *V*sEndA from *Vibrio salmonicida* to saline habitat (Altermark et al., 2008). Thus, halotolerant and halophilic enzymes may have diverse salt-adapted strategies. The halotolerance of lipolytic enzymes can help themselves and the strains producing them well adapt to the saline environments and play a role in marine organic carbon degradation and cycling. It has been reported that halotolerant/halophilic lipolytic enzymes have potentials in industrial processes requiring high salts, low water activity, and the presence of organic solvents.

Based on amino acid sequences and biochemical properties, microbial lipolytic enzymes have been classified into eight families (families I-VIII) (Arpigny and Jaeger, 1999). Enzymes grouped in family V originates from a wide variety of bacteria, including mesophilic, psychrophilic, and thermophilic organisms (Arpigny and Jaeger, 1999). Recently, many members of family V lipolytic enzymes have been discovered (Prive et al., 2013; Sumby et al., 2013; Tchigvintsev et al., 2015). This family contains lipases and esterases, displaying diverse substrate specificities and characteristics (Peng et al., 2011; Chen et al., 2013; Pereira et al., 2015). However, studies on the salt tolerance of this family are still scarce.

Marine environments benefit the discovery of novel enzymes with special characteristics. Because more than 99% of marine microorganisms are still uncultured (Schloss and Handelsman, 2003), metagenomics, a cultivation-independent method, has been developed to discover new functional genes from both cultured and uncultured microorganisms (Handelsman, 2004). The application of functional metagenomics has led to the discovery of several new lipases and esterases from diverse marine environments, such as intertidal flat (Kim et al., 2009), tidal flat sediment (Jeon et al., 2012), and marine surface water (Chu et al., 2008).

To identify novel esterases from marine sediments, in this study, a fosmid library of a deep-sea sediment sample from the South China Sea was constructed, and functional metagenomic screening was performed to obtain novel esterases. A lipolytic enzyme gene *H8* was identified from the library, and the encoding esterase H8 was expressed and characterized. The result showed that H8 was a new member of family V of bacterial lipolytic enzymes with a substrate preference toward short-chain monoesters (C4–C10). H8 displayed high halotolerance. The sequence of H8 contains a large number of basic residues, leading to a high basic/acidic residue ratio and a high predicted isoelectric point (pI). The roles of basic residues in H8 halotolerance were investigated by site-directed mutagenesis. Sequence analysis suggests that H8 together with its homologs represent a new group of halotolerant esterases. These results shed light on marine bacterial esterases and halotolerant enzymes.

## Materials and Methods

### Sample Collection and DNA Extraction

Marine sediment sample S100 was collected from the South China Sea (13.5°N, 118°E) at a water depth of 3,939 m in September 2011. Temperature and salinity of bottom water in this area was 2.4°C and 3.46% (w/v), respectively. The sample was stored at -20°C until processing. Environmental genomic DNA was extracted from the sample by following the SDS-based extraction procedure described by Zhou et al. (1996).

### Metagenomic Library Construction and Screening of Lipolytic Enzymes

The DNA extract was separated by pulsed-filed gel electrophoresis (PFGE), and DNA bands of ∼35 kbp in the gel were extracted by gelase enzymolysis and ethanol precipitation. A metagenomic DNA library was constructed using the CopyControl Fosmid Library Production Kit (Epicentre Biotechnologies, Madison, WI, USA) by following the manufacturer’s instructions. A total of 7,200 fosmid clones were obtained, which were spread onto Luria-Bertani (LB) agar plates supplemented with 1% (v/v) emulsified tributyrin to screen clones with lipolytic enzyme activity. Clones exhibiting a clear zone around the colony were selected to construct their respective subcloning libraries. Fosmid DNA was extracted from positive clones and partially digested by the restriction enzyme Sau3AI. The DNA fragments of 1.5–5 kbp were recovered from an agarose gel, end-repaired and ligated into the pUC19 vector that had been digested by BamHI and pretreated by bacterial alkaline phosphatase. The ligated products were transformed into *E. coli* TOP10 cells, and the transformants were spread onto LB agar plates supplemented with 100 μg/ml ampicillin and 1% (v/v) tributyrin. Transformants forming clear zones were sequenced. The open reading frames (ORFs) in the sequenced DNA fragments were predicted by the GeneMark program^1^ and the genes encoding potential lipolytic enzymes were identified by using the blastx program against the NCBI non-redundant protein database (nr). Multiple sequence alignment was performed using MUSCLE (Edgar, 2004). Phylogenetic analysis was carried out with the neighbor-joining method using MEGA 6.0 (Tamura et al., 2013). The potential signal peptide sequence was predicted by SignalP 4.0 (Petersen et al., 2011).

### Gene Cloning and Protein Expression and Purification

The gene *H8* encoding a lipolytic enzyme was amplified from the fosmid DNA using the primer pair of H8_F (5′-GGGAATTCCATATGCAGTCTGGCACGGTGAG-3′, NdeI digestion site was underlined) and H8_R (5′-CCGCTCGAGCGCCACCGCCGGTTGCGCC-3′, XhoI digestion site was underlined), and cloned into the expression vector pET-22b.

The constructed plasmid pET-22b*-H8* was transformed into *E. coli* BL21 (DE3). Transformants were cultured at 37°C and 180 rpm in LB liquid medium containing 100 μg/mL ampicillin. When the OD_600_ of cells reached approximately 0.6, 1 mM isopropyl-β-D-thiogalactopyranoside (IPTG) was added for the induction of protein expression. Then, the culture was incubated at 20°C and 110 rpm for 20 h. After incubation, the cells in the culture were harvested, resuspended in lysis buffer (50 mM Tris-HCl, 100 mM NaCl, pH 8.0) and disrupted by pressure. The recombinant His-tagged protein in the extract was first purified by Ni affinity chromatography (Qiagen, USA), and further purified by gel filtration chromatography on a Superdex 200 column (GE healthcare, Sweden). Protein concentrations were determined by using the Pierce BCA Protein Assay Kit (Thermo Scientific, USA).

### Enzyme Assays

The esterase activity was measured by monitoring the hydrolysis of *p*-nitrophenyl (*p*NP) esters (Sigma, USA) using a spectrophotometric method (Shirai and Jackson, 1982). The reaction mixture contained 50 mM Tris-HCl buffer (pH 8.0), 0.02 ml of 10 mM substrate, and 0.02 ml enzyme with appropriate concentration in a final volume of 1 m1. After incubation at an indicated temperature for 5 min, the reaction was terminated by an addition of 0.1 ml 20% (w/v) SDS. The absorbance of the reaction mixture at 405 nm was measured to detect the amount of released *p*-nitrophenol (Li et al., 2014). The background hydrolysis of the substrate was determined by using a blank control with a composition identical with the reaction mixture except that the enzyme was replaced by buffer. One unit of enzyme (U) is defined as the amount of enzyme required to liberate 1 μmol *p*-nitrophenol per minute.

### Biochemical Characterization of H8

The substrate specificity of H8 was investigated using the substrates *p*NP acetate (C2), *p*NP butyrate (C4), *p*NP caproate (C6), *p*NP caprylate (C8), *p*NP decanoate (C10), *p*NP laurate (C12), *p*NP myristate (C14), and *p*NP palmitate (C16) (Sigma, USA). The optimum temperature for H8 activity was measured at temperatures ranging from 0 to 60°C at pH 8.0. For thermostability assay, the enzyme was incubated at temperatures ranging from 0 to 60°C for 1 h, and then the residual activity was measured at 35°C. The optimum pH of H8 was determined at 35°C in the Britton–Robinson buffers ranging from pH 4.0 to 13.0. For pH stability assay, the enzyme was incubated in buffers with a pH range of 4.0–13.0 at 25°C for 1 h, and then the residual activity was measured at pH 8.0 and 35°C. The effect of NaCl on H8 activity was determined at NaCl concentrations ranging from 0 to 4.8 M. For salt tolerance assay, the enzyme was incubated at 0°C for 1 h in buffers containing NaCl ranging from 0 to 4.6 M before the residual activity was measured at 35°C.

The effects of metal ions (K^+^, Li^+^, Ba^2+^, Ca^2+^, Co^2+^, Cu^2+^, Fe^2+^, Mg^2+^, Mn^2+^, Ni^2+^, and Zn^2+^) and potential inhibitors (β-mercaptoethanol, DTT, Thiourea, Urea, EDTA, and PMSF) on H8 activity were examined at pH 8.0 and 35°C in a final concentration of 1 mM or 10 mM. The effects of organic solvents on H8 activity were examined using methanol, ethanol, isopropanol, acetone, acetonitrile, dimethylsulfoxide (DMSO), and dimethylformamide (DMF) at final concentrations of 10% and 20% (v/v). The effects of Tween 20, Tween 80, and Triton X-100 on H8 activity were examined at final concentrations of 0.001–0.1% (v/v). The effect of SDS on enzyme activity was measured at final concentrations of 0.001–0.1% (w/v).

### Site-Directed Mutagenesis of H8

Using plasmid pET-22b*-H8* as the template, site-directed mutagenesis on *H8* was performed with the QuikChange^®^ 146 mutagenesis kit II (Agilent technologies, USA) according to the method of QuikChange site-directed mutagenesis (Liu and Naismith, 2008). After verified by DNA sequencing, mutated plasmids were transformed into *E. coli* BL21 (DE3) for protein expression. The purification of H8 mutants was performed under the same conditions as those of the wild type (WT) H8.

### Enzyme Kinetic Assays

Enzyme kinetic assays of H8 and its mutants were carried out at pH 7.5 (50 mM Tris-HCl) using *p*NPC6 at concentrations from 0.02 to 2.0 mM. Kinetic parameters were calculated by non-linear regression fit directly to the Michaelis–Menten equation using the Origin8.5 software.

### Circular Dichroism Spectroscopy

Circular dichroism (CD) spectra of WT H8 and its mutants were recorded at 25°C on a J-810 spectropolarimeter (JASCO, Japan). All the spectra were collected from 200 to 250 nm at a scanning speed of 200 nm/min with a path length of 0.1 cm. Proteins for CD spectroscopy assays were at a concentration of 0.3 mg/ml in 50 mM Tris-HCl buffer (pH 8.0).

### Nucleotide Sequence Accession Number

The nucleotide sequence of H8 has been deposited in the GenBank database under accession number KY273927.

## Results

### Functional Metagenomic Screening of Lipolytic Enzymes

A metagenomic library was constructed from a deep-sea sediment sample from the South China Sea, which contained a total of 7,200 fosmid clones. The metagenomic library represented approximately 252 Mbp environmental DNA assuming an average insert size of 35 kbp. By functional assays, 10 fosmid clones showing lipolytic enzyme activities were screened from this library. The sequences of putative lipolytic enzyme-encoding genes in the identified fosmids were determined by subcloning library construction and subsequent sequencing. Among these genes, the gene *H8* containing 918 bp was predicted to encode a lipolytic enzyme and chosen for further analysis.

### Sequence Analysis of the Lipolytic Enzyme H8

**FIGURE 1:**
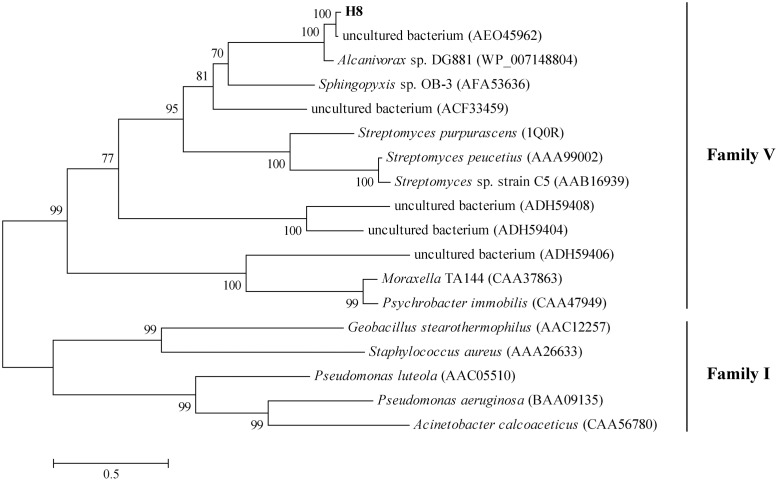
**Phylogenetic tree of esterase H8 and representative lipolytic enzyme sequences from family V**. The tree was built by Neighbor-Joining method with JTT-matrix based model using 218 amino acid positions. Bootstrap analysis of 1000 replicates was executed and values above 50% are shown. The scale for branch length is shown below the tree. Lipases from family I were used as outgroups.

**FIGURE 2:**
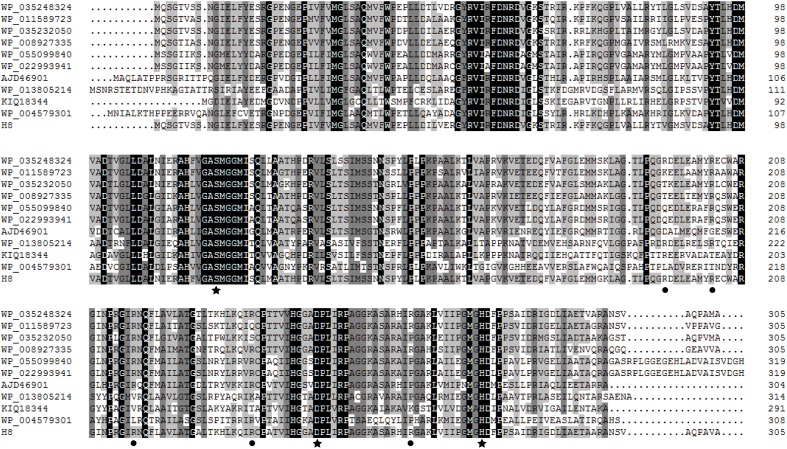
**Multiple sequence alignment of H8 and its homologs**. Identical and similar amino acids are shaded in black and gray, respectively. Stars indicate amino acid residues belonging to the catalytic triad, and circles indicate basic amino acid residues (Arg195, Arg203, Arg216, Arg236, and Arg263) selected for site-directed mutation. Sequence analysis suggested that residues Arg203, Arg216, and Arg236 are highly conserved, and residues Arg195 and Arg263 are partially conserved.

The gene *H8* encodes a lipolytic enzyme of 305 amino acid residues with a predicted molecular weight of 32.8 kDa and a predicted pI of 9.09. Prediction by SignalP 4.0 suggested that H8 may lack an N-terminal signal sequence. Among the characterized lipolytic enzymes, H8 showed the highest sequence identity (46%) to a family V esterase (Est16) from a microbial consortium specialized for diesel oil degradation (Pereira et al., 2015). Phylogenetic tree also showed that H8 belongs to the family V of bacterial lipolytic enzymes (**Figure 1**). Based on sequence alignments with other proteins from family V, the catalytic triad of H8 was identified, which is composed of Ser120, Asp247, and His275 (**Figure 2**). The catalytic Ser120 is located in the conserved GASMGGMI motif, Asp247 in the conserved DPL motif, and His275 in the conserved MG/AHD motif.

### Expression and Characterization of the Esterase H8

**FIGURE 3:**
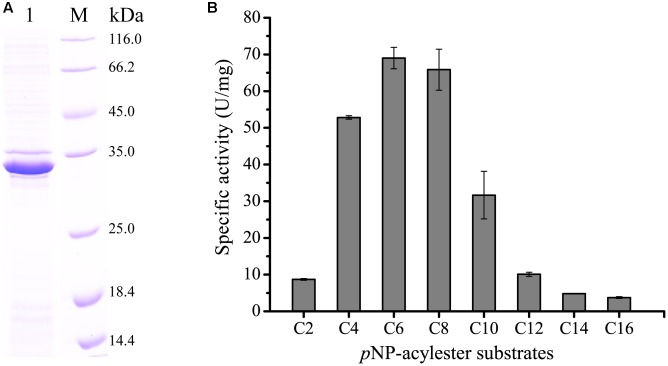
**Sodium dodecyl sulfate polyacrylamide gel electrophoresis (SDS-PAGE) analysis of purified esterase H8 and the substrate specificity of H8**. **(A)** SDS-PAGE analysis of purified H8. Lane 1, purified H8; lane M, protein mass markers. **(B)** Substrate specificity of H8 evaluated with *p*NP esters. The graph shows data from triplicate experiments (mean ± SD).

**FIGURE 4:**
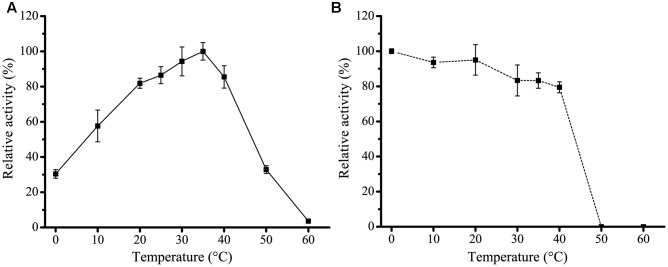
**Effect of temperature on the activity and stability of H8**. **(A)** Effect of temperature on H8 activity. The highest activity of H8 at 35°C (69.2 U/mg) was taken as 100%. **(B)** Effect of temperature on the stability of H8. The enzyme was incubated at 0–60°C for 1 h. The remaining activity was measured under optimal conditions. The activity at 0°C (66.4 U/mg) was taken as 100%. The graphs show data from triplicate experiments (mean ± SD).

**FIGURE 5:**
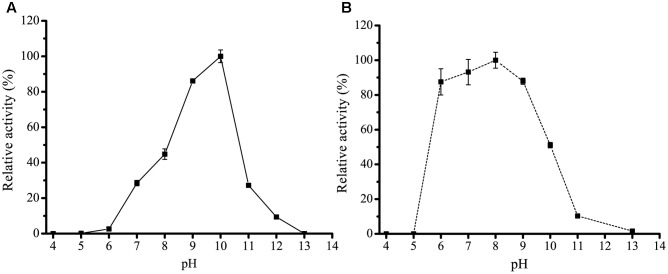
**Effect of pH on the activity and stability of H8**. **(A)** Effect of pH on the activity of H8. The activity was measured at 35°C in Britton–Robinson buffers ranging from pH 4.0 to 13.0. The highest activity at pH 10.0 (70.2 U/mg) was taken as 100%. **(B)** Effect of pH on the stability of H8. The enzyme was incubated in buffers ranging from pH 4.0 to 13.0 at 25°C for 1 h. The remaining activity was measured under optimal conditions. The highest activity at pH 8.0 (54.4 U/mg) was taken as 100%. The graphs show data from triplicate experiments (mean ± SD).

H8 was over-expressed in *E. coli* BL21 (DE3), and the recombinant H8 protein was first purified by Ni affinity chromatography and then by gel filtration chromatography. Sodium dodecyl sulfate polyacrylamide gel electrophoresis (SDS-PAGE) analysis showed that the purified H8 displayed an apparent molecular weight of approximately 33 kDa, accordant to that predicted from its sequence (32.8 kDa) (**Figure 3A**). H8 could efficiently hydrolyze short-chain *p*NP esters (C4–C10), with the maximal activity toward *p*NPC6 (69.0 U/mg) (**Figure 3B** and Supplementary Figure 1). H8 showed a limited ability to degrade *p*NP esters longer than 10 carbon atoms, indicating that H8 is an esterase. H8 showed the highest activity at 35°C and retained 30% of its highest activity at 0°C (**Figure 4A**). H8 retained more than 80% of its highest activity after 1 h incubation at temperatures lower than 40°C, but lost all the activity after 1 h incubation at 50°C (**Figure 4B**). H8 had the highest activity at pH 10.0 (**Figure 5A**) and showed good tolerance in a range of pH 6.0–9.0, retaining over 80% of its highest activity after 1 h incubation in the buffers of pH 6.0–9.0 at 25°C (**Figure 5B**).

The effect of metal ions on the activity of H8 was also investigated (**Table 1**). H8 activity was almost unaffected by K^+^, Li^+^, Ca^2+^, Co^2+^ or Mg^2+^ at 1∼10 mM, but significantly inhibited by Zn^2+^ at 1∼10 mM, and Ba^2+^, Mn^2+^, and Ni^2+^ at 10 mM, and fully inhibited by Cu^2+^ and Fe^2+^ at 10 mM. EDTA had no effect on H8 activity, suggesting that the catalysis by H8 may not require metal ions. H8 activity was significantly inhibited by 10 mM PMSF, indicating that H8 is most likely a serine hydrolase. H8 activity was also severely inhibited by reductants DTT and β-mercaptoethanol. However, H8 showed high resistance to chaotropic agents urea and thiourea (**Table 1**). H8 activity was slightly increased by 0.001–0.1% (v/v) Tween 20, but fully inhibited by 0.01% (w/v) SDS (**Table 2**). Among all the tested organic solvents at 10% (v/v) concentration, methanol, ethanol, DMF, and DMSO slightly increased H8 activity, and other detergents slightly reduced H8 activity. At 20% (v/v) concentration, DMF and DMSO had nearly no effect on H8 activity, whereas other detergents significantly inhibited H8 activity (**Table 3**).

**Table 1:** Effects of metal ions and potential inhibitors on H8 activity.

Compound	Relative/Residual activity (%)	
	1 mM	10 mM
K^+^	107.8 ± 5.8	107.6 ± 1.5
Li^+^	99.8 ± 2.1	86.1 ± 3.1
Ba^2+^	93.4 ± 4.4	59.4 ± 2.8
Ca^2+^	94.9 ± 8.1	90.9 ± 6.1
Co^2+^	84.9 ± 0.8	74.3 ± 2.6
Cu^2+^	82.2 ± 2.7	0.7 ± 0.2
Fe^2+^	85.0 ± 1.4	LD^a^
Mg^2+^	96.8 ± 6.1	84.1 ± 5.4
Mn^2+^	76.0 ± 3.3	57.2 ± 2.6
Ni^2+^	67.5 ± 1.8	57.1 ± 1.7
Zn^2+^	25.1 ± 0.6	11.5 ± 0.4
β-Mercaptoethanol	78.6 ± 3.1	11.9 ± 2.8
DTT	31.3 ± 1.2	5.0 ± 2.7
Thiourea	111.5 ± 1.2	118.2 ± 5.1
Urea	103.5 ± 8.0	115.2 ± 4.0
EDTA	106.8 ± 1.8	114.4 ± 1.3
PMSF	96.2 ± 4.7	33.4 ± 1.1

^a^*LD indicates that the value was less than the limit of detection*.

**Table 2:** Effects of detergents on H8 activity.

Detergent	Relative activity (%)		
	0.001% (v/v)	0.01% (v/v)	0.1% (v/v)
Tween 20	128.9 ± 5.6	112.3 ± 4.9	110.6 ± 3.4
Tween 80	102.9 ± 3.4	103.8 ± 3.0	112.3 ± 2.7
Triton X-100	113.4 ± 1.8	84.2 ± 0.5	62.8 ± 3.0
SDS^a^	33.0 ± 0.6	1.4 ± 0.2	LD^b^

^a^*The concentrations of SDS used were presented in w/v*.

^b^*LD indicates that the value was less than the limit of detection*.

**Table 3:** Effects of organic solvents on H8 activity.

Organic solvent	Relative activity (%)		
	10% (v/v)	20% (v/v)
Methanol	122.5 ± 4.6	52.6 ± 2.2
Ethanol	120.4 ± 8.6	24.4 ± 0.9
Isopropanol	70.2 ± 6.1	4.4 ± 0.3
Acetone	82.3 ± 3.0	16.2 ± 0.5
Acetonitrile	65.6 ± 3.5	2.7 ± 0.3
DMF	109.9 ± 3.5	75.4 ± 1.9
DMSO	110.2 ± 2.1	104.7 ± 1.9

### High Salt Tolerance of H8

**FIGURE 6:**
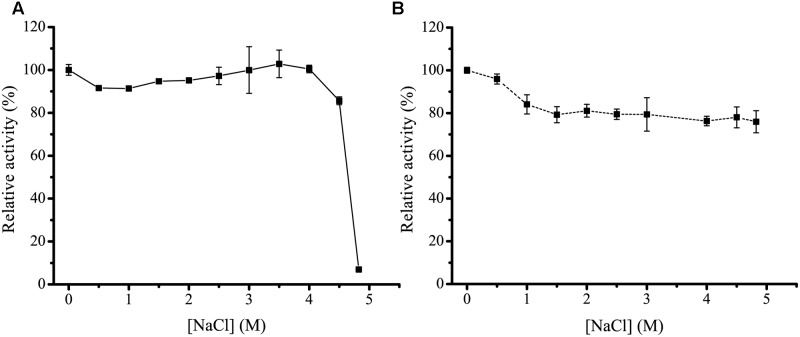
**Effect of NaCl on the activity and stability of H8**. **(A)** Effect of NaCl on the activity of H8. The activity was measured at 35°C in 50 mM Tris-HCl buffer with different concentrations of NaCl. The activity in 0 M NaCl (70.9 U/mg) was taken as 100%. **(B)** Effect of NaCl on the stability of H8. The enzyme was incubated at 0°C for 1 h in buffers containing NaCl ranging from 0 to 4.6 M. The remaining activity was measured under optimal conditions. The activity in 0 M NaCl (68.6 U/mg) was taken as 100%. The graphs show data from triplicate experiments (mean ± SD).

Because the *H8* gene is isolated from a deep-sea sediment, we investigated the effect of NaCl of different concentrations on the activity and stability of H8. H8 still had full activity in NaCl with a concentration as high as 4.0 M (**Figure 6A**), indicating that H8 has high salt tolerance. Moreover, after 1 h incubation in 4.6 M NaCl, H8 still retained 80% activity (**Figure 6B**). These results show that H8 is a halotolerant enzyme.

### High Contents of Basic Residues in H8

Unlike most reported halophilic/halotolerant enzymes that have high acidic/basic residue ratios and relatively low pI values ranging from 4.3 to 6.8 (Ng et al., 2000; Kennedy et al., 2001; Dassarma et al., 2013), H8 contains more basic residues (10.49%) than acidic residues (9.18%) (**Table 4**), leading to a high predicted pI value of 9.09. By searching NCBI nr database using the H8 sequence as a query, more than 10 homologs of H8 are found to have more basic residues than acidic residues and high pI values (**Table 4**), suggesting that H8 and its homologs may represent an uncharacterized group of lipolytic enzymes rich in basic residues.

**Table 4:** Comparison of the amino acid composition of H8 and its homologs and reported halotolerant enzymes.

	Halotolerant H8	H8 homologs	Halophilic *V*sEndA	Halotolerant PE10	Halophilic Hm EST
pI value	9.09	7.96–10.28	9.57	4.65	4.24
Arg + Lys (%)	10.49	10.00–11.84	17.06	6.45	5.8
Asp + Glu (%)	9.18	8.20–9.97	9.48	10.75	16.8
(Arg + Lys)/(Asp + Glu)	1.14	1.03–1.38	1.80	0.60	0.35
Sequence identity to H8^a^	100%	44–99%	–	–	–

^a^*–, no sequence identity detected*.

### The Role of Basic Residues in the Salt Tolerance of H8

Until now, only one halophilic enzyme, the endonuclease *Vs*EndA from *V. salmonicida*, is found to have an overwhelming number of basic residues distributed on the protein surface for its haloadaption (Altermark et al., 2008). To study the roles of the basic residues, especially the surface basic residues, in the salt tolerance of H8, we tried to obtain its crystal structure or modeled structure. Unfortunately, the crystal structure of H8 was unable to be solved due to the low resolution of the H8 crystals we obtained. In addition, due to the low sequence identity (lower than 28%) between H8 and reported proteins with resolved structures, no modeled structure of H8 could be constructed. Finally, according to multiple sequence alignment (**Figure 2**), we selected five basic residues (Arg195, Arg203, Arg216, Arg236, and Arg263) for site-directed mutation to acidic Glu to investigate their roles in H8 halotolerance. Residues Arg195 and Arg263 are partially conserved and residues Arg203, Arg216, and Arg236 are highly conserved in H8 homologs (**Figure 2**).

**FIGURE 7:**
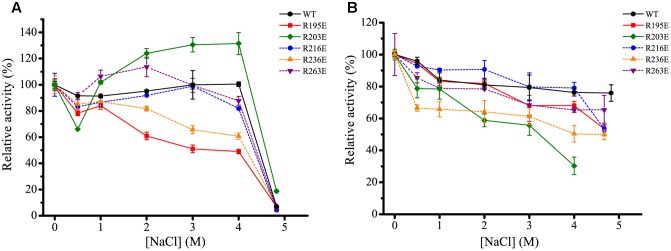
**Effect of NaCl on the activity and stability of the mutants of H8**. **(A)** Effect of NaCl on the activity of WT H8 and its mutants. The activities of H8 and its mutants were determined at different NaCl concentrations at their respective optimum temperatures. The activities of WT H8 (70.9 U/mg), R195E (65.6 U/mg), R203E (7.5 U/mg), R216E (82.6 U/mg), R236E (53.0 U/mg), and R263E (62.0 U/mg) in 0 M NaCl were taken as 100%, respectively. **(B)** Effect of NaCl on the stability of WT H8 and its mutants. The enzymes were incubated in buffers containing different NaCl concentrations at 0°C for 1 h, and the residual activity was measured at their optimum temperatures, respectively. The activities of WT H8 (68.6 U/mg), R195E (64.5 U/mg), R203E (6.7 U/mg), R216E (72.8 U/mg), R236E (48.8 U/mg), and R263E (61.0 U/mg) in 0 M NaCl were taken as 100%, respectively. The graphs show data from triplicate experiments (mean ± SD).

**FIGURE 8:**
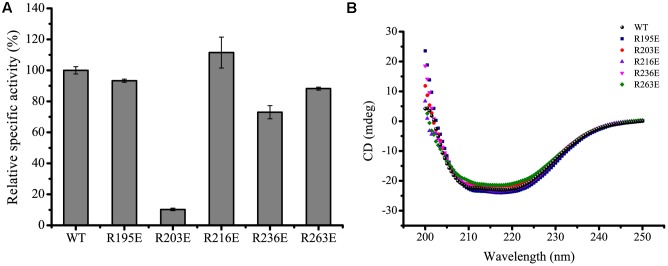
**Relative specific activities and circular dichroism (CD) spectra of WT H8 and its mutants**. **(A)** Relative specific activities of WT H8 and its mutants. The specific activity of WT H8 (69.7 U/mg) was defined as 100%. **(B)** CD spectra of WT H8 and its mutants. All the spectra were collected from 200 to 250 nm at a scanning speed of 200 nm/min with a path length of 0.1 cm. The graphs show data from triplicate experiments (mean ± SD).

The effect of NaCl on the activities and stabilities of the mutants was measured and compared to WT H8 (**Figure 7**). Under their respective optimum temperatures, the effect of NaCl on the activities and stabilities of mutants R216E and R263E was similar to that of the WT (**Figures 7A,B**), suggesting that these two residues Arg216 and Arg263 may not be related to the salt tolerance of H8. In addition, mutants R216E and R263E had similar *K*_m_ and *k*_cat_ values and specific activities to the WT (**Figure 8A** and **Table 5**), indicating that these two mutations had little effect on the substrate binding and catalysis of H8 and that Arg216 and Arg263 are potentially surface residues.

**Table 5:** Impact of mutations on H8 stability and activity.

Enzyme	*T*_opt_ (°C)	*V*_max_ (μM/min/mg)	*K*_m_ (mM)	*k*_cat_ (s^-1^)	*k*_cat_/*K*_m_ (mM^-1^ s^-1^)
WT	35	73.36 ± 1.91	0.074 ± 0.014	40.10 ± 1.04	539.8 (100%)
R195E	30	51.96 ± 2.92	0.052 ± 0.011	28.41 ± 1.59	546.2 (101.2%)
R203E	30	8.50 ± 1.09	0.070 ± 0.002	4.64 ± 0.59	66.6 (12.3%)
R216E	35	74.49 ± 2.63	0.072 ± 0.008	40.72 ± 2.44	546.7 (101.3%)
R236E	30	46.53 ± 1.54	0.053 ± 0.003	25.44 ± 0.84	481.8 (89.3%)
R263E	35	50.01 ± 2.37	0.058 ± 0.008	26.64 ± 1.19	459.3 (85.1%)

Although the activity of mutant R203E was slightly stimulated by 1.2- to 1.3-fold in NaCl ranging from 2.0 to 4.0 M, its stability was significantly reduced in increased concentrations of NaCl. After incubated in 4.0 M NaCl for 1 h, H8 retained 80% activity, whereas R203E retained only 30% activity, indicating that mutant R203E is less tolerant than the WT under high salts. Mutation R203E had no impact on the *K*_m_ of H8, but significantly reduced its *k*_cat_ and specific activity (**Figure 8A** and **Table 5**). These data suggest that Arg203 might be directly or indirectly involved in the catalysis of H8.

The effect of NaCl on the stability of mutant R195E was similar to that of the WT, whereas its activity was significantly reduced by NaCl. Compared to WT H8, the activity of mutant R195E in 4 M NaCl was reduced by 51%, suggesting its potential role in the salt tolerance of H8. Mutation R195E had no impact on the specific activity of H8 and small impact on the *K*_m_ and *k*_cat_ values (**Figure 8A** and **Table 5**), indicating that Arg195 is potentially located on the surface of H8 protein.

For mutant R236E, both the activity and stability was significantly reduced in increased concentrations of NaCl, suggesting that Arg236 may play an important role in the salt tolerance of H8. Mutant R236E had only small effect on the substrate binding and catalysis of H8 (**Table 5**), suggesting that residue Arg236 is potentially a surface residue.

Circular dichroism spectral analysis showed that these mutations caused no visible changes in H8 structure (**Figure 8B**), indicating that the decrease in the enzymatic activity and stability of the mutants resulted from residue substitution rather than structural changes.

## Discussion

In this study, a metagenomic library containing 7,200 fosmid clones was constructed from a deep-sea sediment sample from the South China Sea to functionally screen lipolytic enzyme-encoding genes, and a gene encoding an esterase H8 was identified, cloned and over-expressed. Phylogenetic analysis showed that H8 belongs to the family V of bacterial lipolytic enzymes. Among the characterized lipolytic enzymes, H8 has the highest identity (46%) to Est16 from a microbial consortium specialized for diesel oil degradation (Pereira et al., 2015). However, H8 shows different substrate specificities from Est16 (Pereira et al., 2015). H8 can efficiently hydrolyze short-chain monoesters (C4–C10), especially for *p*NPC6 and *p*NPC8, while Est16 prefers to hydrolyze *p*NPC4 and *p*NPC5 (Pereira et al., 2015). Therefore, H8 is a new family V esterase.

A few halotolerant/halophilic lipolytic enzymes have been reported. Consistent with other known halotolerant/halophilic enzymes, halotolerant/halophilic lipolytic enzymes contain more acidic residues (Asp and Glu) than basic residues in their sequences, leading to their low pI values (Ng et al., 2000; Kennedy et al., 2001; Dassarma et al., 2013). Moreover, modeled structural analysis suggests that a large number of negatively charged acidic residues are distributed over the protein surface of halotolerant/halophilic lipolytic enzymes, which may form a protective solvation shell to keep the protein surface hydrated and promote the adaption of the protein to salinity (De Santi et al., 2016; Wang et al., 2016). Until now, reports on the salt tolerance of family V lipolytic enzymes are still scarce. We studied the effect of NaCl on the activity and stability of H8. The result showed that H8 has high halotolerance. However, unlike most reported halotolerant/halophilic enzymes, H8 contains a large number of basic residues in sequence, leading to its high basic/acidic residue ratio and high pI value (9.09). In addition, more than 10 homologous sequences with similar basic/acidic residue ratios and predicted pI values to H8 are found in NCBI nr database. Therefore, H8 and its homologs may represent a new subgroup of family V halotolerant lipolytic enzymes rich in basic residues.

Up to date, only a halophilic endonuclease *V*sEndA from *V. salmonicida* is reported to contain more basic residues than acidic residues and have high pI value (9.57) (Altermark et al., 2008). Moreover, structural analysis shows that the protein surface of *V*sEndA is populated with positively charged basic residues, which may result in the haloadaption of *V*sEndA (Altermark et al., 2008). We studied the roles of five conserved basic residues in the salt tolerance of H8 by residue replacement. The results suggested that Arg195, Arg203, and Arg236 may play a role in the salt tolerance of H8, but Arg216 and Arg263 have little effect on the salt tolerance of H8. However, due to the lack of H8 structure, it is difficult to determine the exact positions and roles of these basic residues in H8, which still need further study.

In addition, H8 is also a cold-adapted enzyme. H8 had a low optimal temperature (35°C) for activity and still remained 30% of the maximal activity at 0°C. The halotolerant and cold-adapted characteristics indicate that H8 is well adapted to deep-sea sediment and may play a role in marine organic degradation and carbon cycling. Moreover, the good halotolerance of H8 implies its potentials in harsh industrial processes requiring high salts, low water activity, and the presence of organic solvents (such as DMSO).

## Author Contributions

YZ, JH, and Y-QZ performed all experiments. P-YL and X-LC directed the experiments. YZ and P-YL wrote the manuscript. B-BX and MS helped in data analysis. Y-ZZ and B-CZ designed the research.

## Conflict of Interest Statement

The authors declare that the research was conducted in the absence of any commercial or financial relationships that could be construed as a potential conflict of interest.

## Footnotes

1 http://topaz.gatech.edu/GeneMark/
