# Supplementary material for: Identification and Characterization of a Novel Salt-Tolerant Esterase from the Deep-Sea Sediment of the South China Sea
Source: Front Microbiol. 2017 Mar 23;8:441. doi: 10.3389/fmicb.2017.00441 (PMC5362591; doi:10.3389/fmicb.2017.00441)
Supplement: Supplementary file 1 [file Image_1.PDF]

# *Supplementary Material*

# Identification and characterization of a novel salt-tolerant esterase from the deep-sea sediment of the South China Sea

Yi Zhang[1], Jie Hao[1], Yan-Qi Zhang[1], Xiu-Lan Chen[1], Bin-Bin Xie[1], Mei-Shi[1], Bai-Cheng Zhou[1], Yu-Zhong Zhang[1,2], Ping-Yi Li[1,*]

[1]State Key Laboratory of Microbial Technology, Marine Biotechnology Research Center, Institute of Marine Science and Technology, Shandong University, Jinan 250100, China
[2]Laboratory for Marine Biology and Biotechnology, Qingdao National Laboratory for Marine Science and Technology, Qingdao, China

*Address correspondence to: Ping-Yi Li, State Key Laboratory of Microbial Technology, Shandong University, Jinan 250100, P. R. China. Tel: +86-531-88365013; Fax: +86-531-88564326. E-mail: lipingyipeace@sdu.edu.cn

## Supplementary Figure

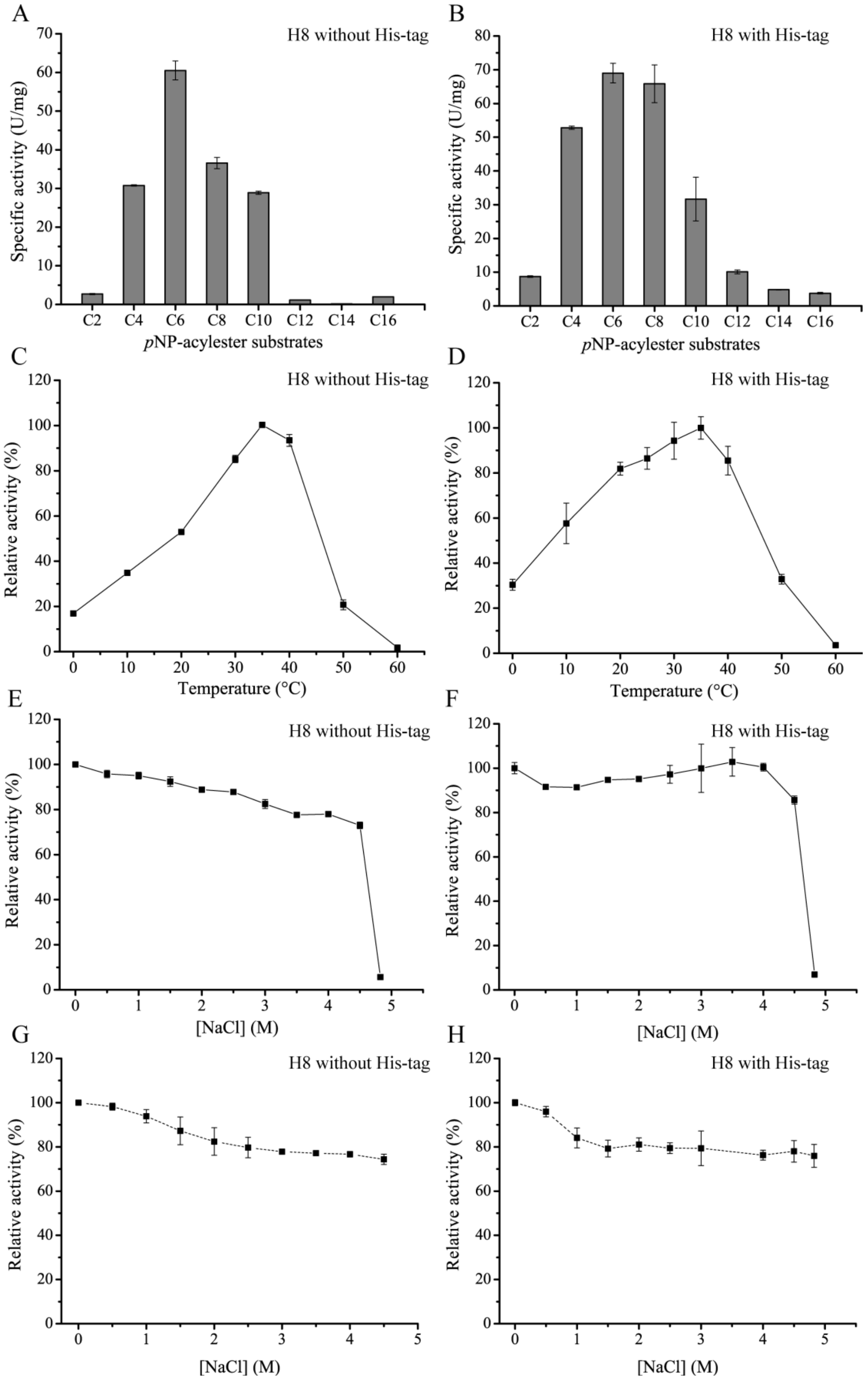


**Supplementary Figure 1. Biochemical analysis of H8 with or without the His-tag.** (A) Substrate specificity of H8 without the His-tag. (B) Substrate specificity of H8 with the His-tag. (C) Effect of temperature on the activity of H8 without the His-tag. The highest activity of H8 (57.6 U/mg) at 35 ℃ was taken as 100%. (D) Effect of temperature on the

activity of H8 with the His-tag. The highest activity of H8 (69.0 U/mg) at 35 ℃ was taken as 100%. (E) Effect of NaCl on the activity of H8 without the His-tag. The activity in 0 M NaCl (55.0 U/mg) was taken as 100%. (F) Effect of NaCl on the activity of H8 with the His-tag. The activity in 0 M NaCl (70.9 U/mg) was taken as 100%. (G) Effect of NaCl on the stability of H8 without the His-tag. The activity in 0 M NaCl (49.9 U/mg) was taken as 100%. (H) Effect of NaCl on the stability of H8 with the His-tag. The activity in 0 M NaCl (68.6 U/mg) was taken as 100%.
